# The Neutral Self-Assembling Peptide Hydrogel SPG-178 as a Topical Hemostatic Agent

**DOI:** 10.1371/journal.pone.0102778

**Published:** 2014-07-21

**Authors:** Seiji Komatsu, Yusuke Nagai, Keiji Naruse, Yoshihiro Kimata

**Affiliations:** 1 Department of Plastic and Reconstructive Surgery, Okayama Saiseikai General Hospital, Okayama, Japan; 2 Department of Cardiovascular Physiology, Okayama University Graduate School of Medicine, Dentistry and Pharmaceutical Sciences, Okayama, Japan; 3 Menicon Co., Ltd., Nagoya, Japan; 4 Department of Plastic and Reconstructive Surgery, Okayama University Graduate School of Medicine, Dentistry and Pharmaceutical Sciences, Okayama, Japan; National Cheng Kung University, Taiwan

## Abstract

Conventional self-assembling peptide hydrogels are effective as topical hemostatic agents. However, there is a possibility to harm living tissues due to their low pH. The aim of the present study was to demonstrate the efficacy of SPG-178, a neutral self-assembling peptide hydrogel, as a topical hemostatic agent. First, we measured the bleeding duration of incisions made on rat livers after application of SPG-178 (1.0% w/v), SPG-178 (1.5% w/v), RADA16 (1.0% w/v), and saline (n = 12/group). Second, we observed the bleeding surfaces by transmission electron microscopy immediately after hemostasis. Third, we measured the elastic and viscous responses (G′ and G″, respectively) of the hydrogels using a rheometer. Our results showed that bleeding duration was significantly shorter in the SPG-178 group than in the RADA16 group and that there were no significant differences in transmission electron microscopy findings between the groups. The greater the G′ value of a hydrogel, the shorter was the bleeding duration. We concluded that SPG-178 is more effective and has several advantages: it is non-biological, transparent, nonadherent, and neutral and can be sterilized by autoclaving.

## Introduction

Hemostasis is one of the most important aspects in surgery because intraoperative blood loss increases the incidence of postoperative morbidity and mortality [1], [2]. Currently, various biological and synthetic topical agents shown to facilitate intraoperative hemostasis are commercially available. Biological hemostatic agents include gelatin products, oxidized cellulose, microfibrillar collagen, topical thrombin, fibrin sealants, and platelet gels, whereas cyanoacrylate is a commonly applied synthetic hemostatic agent. Thus, many topical hemostatic agents are available. However, there are several problems [3], [4]. For example, most of sheet- and powder-type topical hemostatic agents are opaque. Therefore, it is almost impossible to confirm the completion of hemostasis by observing the bleeding surface through these agents. The use of biological topical hemostatic agents, which are often administered during surgery, could cause risks of inducing allergic reactions and unknown infectious diseases [5], [6].

To solve these problems, the use of self-assembling peptide hydrogels has been proposed [7]. The complete sequence of a self-assembling peptide was originally found in a region of alternating hydrophobic and hydrophilic residues in a yeast protein, zuotin [8], which was characterized by a stable β-sheet structure and known to undergo self-assembly into nanofibers that could further form a hydrogel [9], [10]. Self-assembling peptide hydrogels have already been employed for topical hemostasis [7], [11]–[13], tissue engineering [14]–[17], drug delivery systems [18], [19], and wound healing [20], [21].

RADA16 (RADARADARADARADA; R =  arginine, A =  alanine, and D =  aspartic acid, BD Biosciences, San Jose, CA, USA) is a representative self-assembling peptide hydrogel, the use of which as a topical hemostatic agent has already been reported [7], [13]. However, because of its very low pH (approximately 3–4), untreated RADA16 retains the potential to harm living tissues. Therefore, to avoid cell necrosis and receive the full benefit of the self-assembling peptide technology, the pre-neutralization procedure was often employed [22]–[29]. In one particular case, it took a week to gradually neutralize the hydrogel by a solvent substitution though the neutralized RADA16 successfully demonstrate its capability to bridge the injured spinal cord of rats [29]. In addition, the hydrogel structure of RADA16 is unstable under physiological condition. After the neutralization procedure, the hydrogel tends to break under mechanical stress [22], [30]. The RADA16 peptide molecule has the same number of positive and negative charges. The isoelectric point is 6.1 [31], so that its net charge is nearly zero at physiological pH conditions (approximately pH 7). The peptide's solubility is accordingly very low. Therefore, it can easily precipitate or break. In general, other conventional self-assembling peptide hydrogels have similar problems.

For those reasons, we considered using the neutral (pH = 6.5–7.5) self-assembling peptide hydrogel SPG-178 (Self-assembling Peptide Gel, amino acid sequence #178; [CH_3_CONH] -RLDLRLALRLDLR-[CONH_2_]; R =  arginine, L =  leucine, D =  aspartic acid, and A =  alanine, Menicon Co., Ltd., Nagoya, Japan), as a hemostatic agent. The SPG-178 peptide is synthesized through a chemical process. The peptide has been shown to form an antiparallel β-sheet structure as its secondary structure in aqueous solution. The peptide self-assembles to form nanofibers of <10-nm diameter that can further form a stable net structure. The results of three-dimensional cell culture have shown that SPG-178 is noncytotoxic. The isoelectric point of SPG-178 was designed to be 11.5 by employing four cationic arginine and two aspartic acid residues for better stability in neutral pH environment (around pH = 7) [32]. The aim of the present study was to investigate the efficacy of SPG-178 as a topical hemostatic agent in a rat liver laceration model. We also compared the SPG-178 and RADA16 hydrogels using transmission electron microscopy (TEM) and rheology measurements.

## Materials and Methods

The SPG-178 (1.0% and 1.5% w/v) and RADA16 (1.0% w/v) hydrogels were purchased from Menicon Co., Ltd. and BD Biosciences (San Jose, CA, USA), respectively.

### Ethics Statement

All experimental rats were housed in a climate-controlled facility and food (commercial pellets for experimental animals) and water was available ad libitum. All animal experiments were performed in strict accordance with the guidelines of the National Institutes of Health (Bethesda, MD, USA) and were performed at the Department of Animal Resources, Advanced Science Research Center, Okayama University (Okayama, Japan) after receipt of approval from the Animal Care and Use Committee, Okayama University (permit number OKU-2012239).

### Bleeding Duration in a Rat Liver Laceration Model

**Figure 1:**
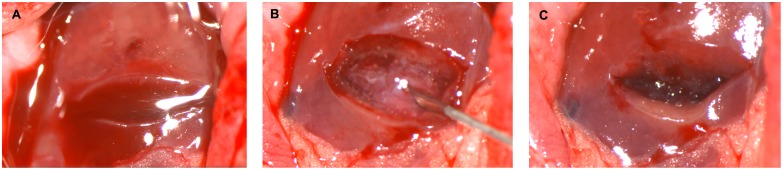
Hemostasis in a rat liver laceration model. (A) Ventral, inferior, horizontal incisions were made with a scalpel on the left hepatic lobe. (B) Bleeding decreased immediately following application of the 1.5% (w/v) SPG-178 hydrogel to the bleeding surface. Because SPG-178 is transparent, the bleeding surface was visible. (C) SPG-178 was wiped and washed away with saline 10 min later, but rebleeding did not occur.

A total of 48 male Wistar rats (8 weeks of age) were randomly divided into four groups of 12 rats each: (1) SPG-178 (1.0% w/v), (2) SPG-178 (1.5% w/v), (3) RADA16 (1.0% w/v), and (4) a saline group (control). All rats were anesthetized by intraperitoneal injection of pentobarbital sodium at a dose of 50 mg/kg and each was placed in the supine position to open the abdomen to expose the liver. Horizontal 10-mm incisions, 3 mm deep, were made with a scalpel on the front of the lower part of the left lobe of the liver, 100 µL of hydrogel or saline was applied to the bleeding surface using a syringe fitted with a 27-G needle, and bleeding duration was measured. The area was wiped and then washed with saline 10 min later (Fig. 1) and the abdominal incision was closed with sutures.

On postoperative day 7, the rats were anesthetized by intraperitoneal injection of pentobarbital sodium at a dose of 50 mg/kg and the abdomen of each was opened to expose the liver. Hepatectomy was then performed for histopathological examination. The livers were fixed in 10% (w/v) formalin neutral buffer solution for 5 days, dehydrated with graded concentrations of ethanol, which was replaced with xylene, embedded in paraffin, cut into 4.5-µm sections with a microtome, and plated on slides, which were stained with hematoxylin and eosin and Masson's trichrome stain. The residual hydrogels and foreign body reaction were examined under a light microscope. After hepatectomy, all rats were sacrificed by intraperitoneal injection of pentobarbital sodium at a dose of 120 mg/kg.

Statistical analysis was performed using Statistical Package for Social Sciences software (SPSS Inc., Chicago, IL, USA). Intergroup differences were identified by one-way analysis of variance using the post-hoc Tukey's honestly significant difference test to allow for multiple comparisons. A probability (*p*) value of <0.01 was considered statistically significant. All data are presented as the mean ± standard error of the mean.

### TEM of Bleeding Liver Surfaces

As described above, all rats were anesthetized and hydrogels (SPG-178, 1.5% w/v; RADA16, 1.0% w/v, respectively; n = 2 each) were applied to the lacerated livers. Hepatectomy was performed 10 min later. However, the hydrogels were not wiped clear to allow observation. After hepatectomy, all rats were sacrificed by intraperitoneal injection of pentobarbital sodium at a dose of 120 mg/kg. The livers were excised, fixed at 4°C overnight in 0.1 M phosphate buffer containing 2% (v/v) glutaraldehyde and 2% (v/v) paraformaldehyde, postfixed in 2% (w/v) osmium tetroxide solution for 1.5 h at room temperature, dehydrated using graded concentrations of ethanol, embedded in Spurr resin (Polysciences, Inc., Warrington, PA, USA), cut into ultrathin (60–90 nm) sections using a Leica EM UC6 ultramicrotome (Leica, Vienna, Austria), and plated on slides, which were stained with uranyl acetate and lead citrate, and then visualized using a Hitachi 7650 electron microscope (Hitachi, Tokyo, Japan; Central Research Laboratory, Okayama University Medical School) operated at 80 kV. The sections were then stained with toluidine blue stain to determine the area of interest.

### Rheology Measurement of Hydrogels

Rheology measurements were performed for the hydrogels (SPG-178, 1.0% w/v; SPG-178, 1.5% w/v; and RADA16, 1.0% w/v). In brief, a 40-µL aliquot of the hydrogels was placed on the plate of a rheometer (AR1000, TA Instruments, New Castle, DE, USA) and a 20-mm-diameter, 1° aluminum cone with truncation at 24 µm was then lowered so that the tip was 24 µm above the plate. A solvent trap was used to maintain a water-saturated atmosphere to prevent evaporation of the solvent during the measurements. The hydrogels were tested over a range of frequencies of 10–0.1 rad/s at 1.0-µNm oscillatory torque to measure the storage modulus (G′, the elastic response) and the loss modulus (G″, the viscous response) at 37°C.

## Results

### Bleeding Duration in the Rat Liver Laceration Model

**Figure 2:**
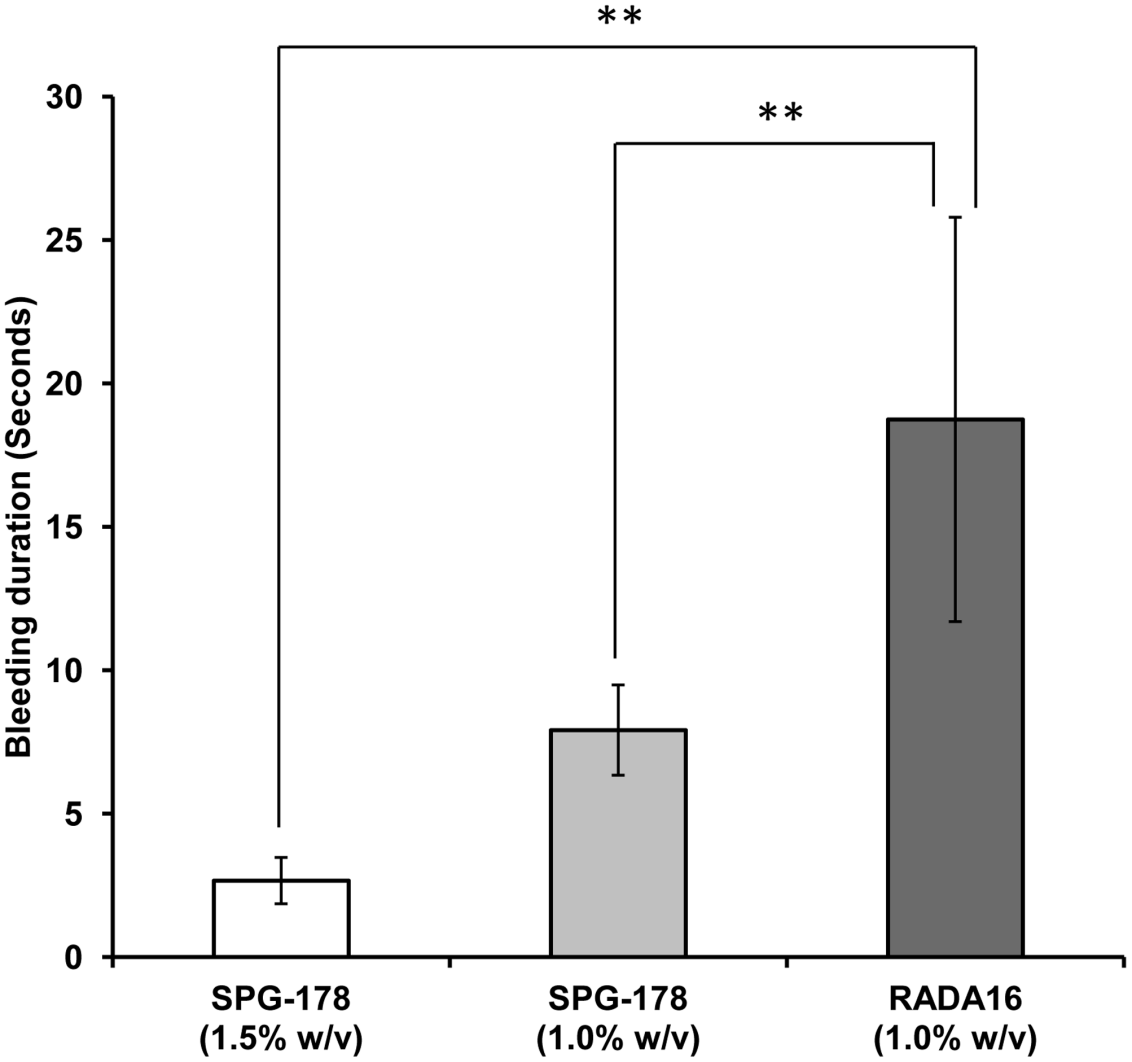
Bleeding durations in a rat liver laceration model. Bleeding durations were 2.7±0.8, 7.9±1.6, and 18.8±7.0 in the 1.5% (w/v) SPG-178, 1.0% (w/v) SPG-178, and 1.0% (w/v) RADA16 groups, respectively. Bleeding duration was significantly shorter in the SPG-178 (1.0% and 1.5% w/v) groups than in the 1.0% (w/v) RADA16 group (**p<0.01; n = 12; one-way analysis of variance with post-hoc Tukey's honestly significant difference test). Bars represent means ± standard errors of the mean.

The bleeding from liver incisions decreased immediately following application of either hydrogel to the bleeding surface. The bleeding surface was also visible with the application of both hydrogels (Fig. 1, Video S1). The bleeding durations were 2.7±0.8, 7.9±1.6, 18.8±7.0, and 267.1±63.4 s for the SPG-178 (1.5% w/v), SPG-178 (1.0% w/v), RADA16 (1.0% w/v), and saline groups, respectively (Fig. 2). The bleeding duration was significantly shorter in the SPG-178 groups (1.0% w/v and 1.5% w/v) than in the RADA16 (1.0% w/v) group (*p* = 0.009). Rebleeding attributable to removal of the hydrogels was not observed in any rat. No rats died prior to being euthanized.

**Figure 3:**
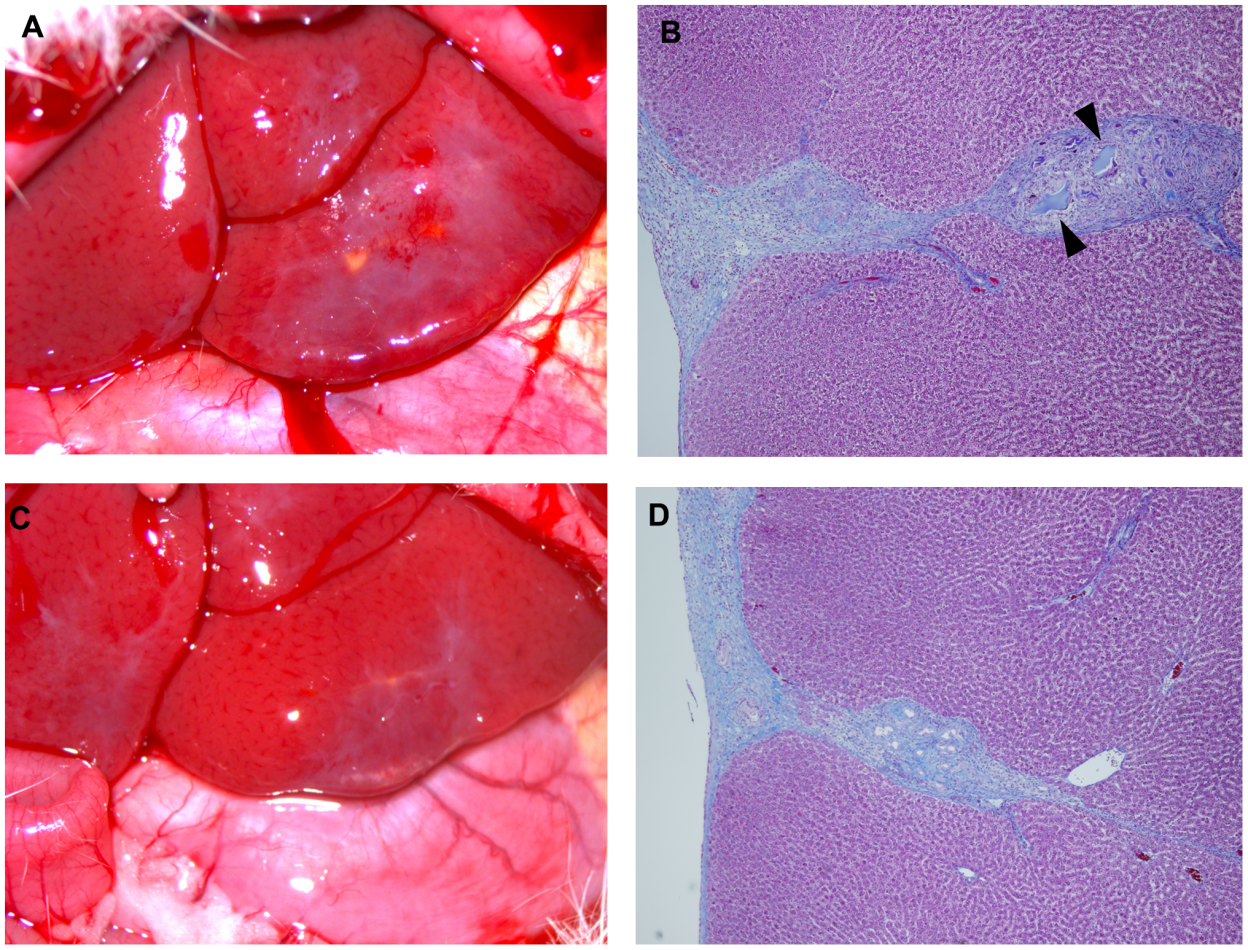
Rat liver lacerations on postoperative day 7. (A) Healed liver lacerations in the 1.5% (w/v) SPG-178 group. (B) A liver laceration replaced with scar tissue. Pieces of SPG-178 (arrowheads) are identifiable in the scar tissue, but were invisible to the naked eye. Macrophages and some foreign body giant cells surrounding SPG-178 were observed, but there was no strong immune reaction against a foreign body. (C, D) A representative figure of a healed liver laceration in the saline group. No significant differences were observed between the 1.5% (w/v) group and the saline group. All slides were stained with Masson's trichrome. Magnification, ×100 (B, D).

On postoperative day 7, the liver lacerations in all rats were healed (Fig. 3) and no significant differences on gross liver inspection were detected between the four groups. Liver lacerations were replaced with scar tissue in all cases. Remnants of the hydrogels were identified in the scar tissue by histopathological examination, but were invisible to the naked eye. Macrophage infiltration and some foreign-body giant cells surrounding hydrogel remnants were observed, but there was no evidence of a strong foreign-body immune response. No differences were observed between the SPG-178 and RADA16 groups on histopathological examination of the liver tissues.

### TEM of Bleeding Liver Surfaces

**Figure 4:**
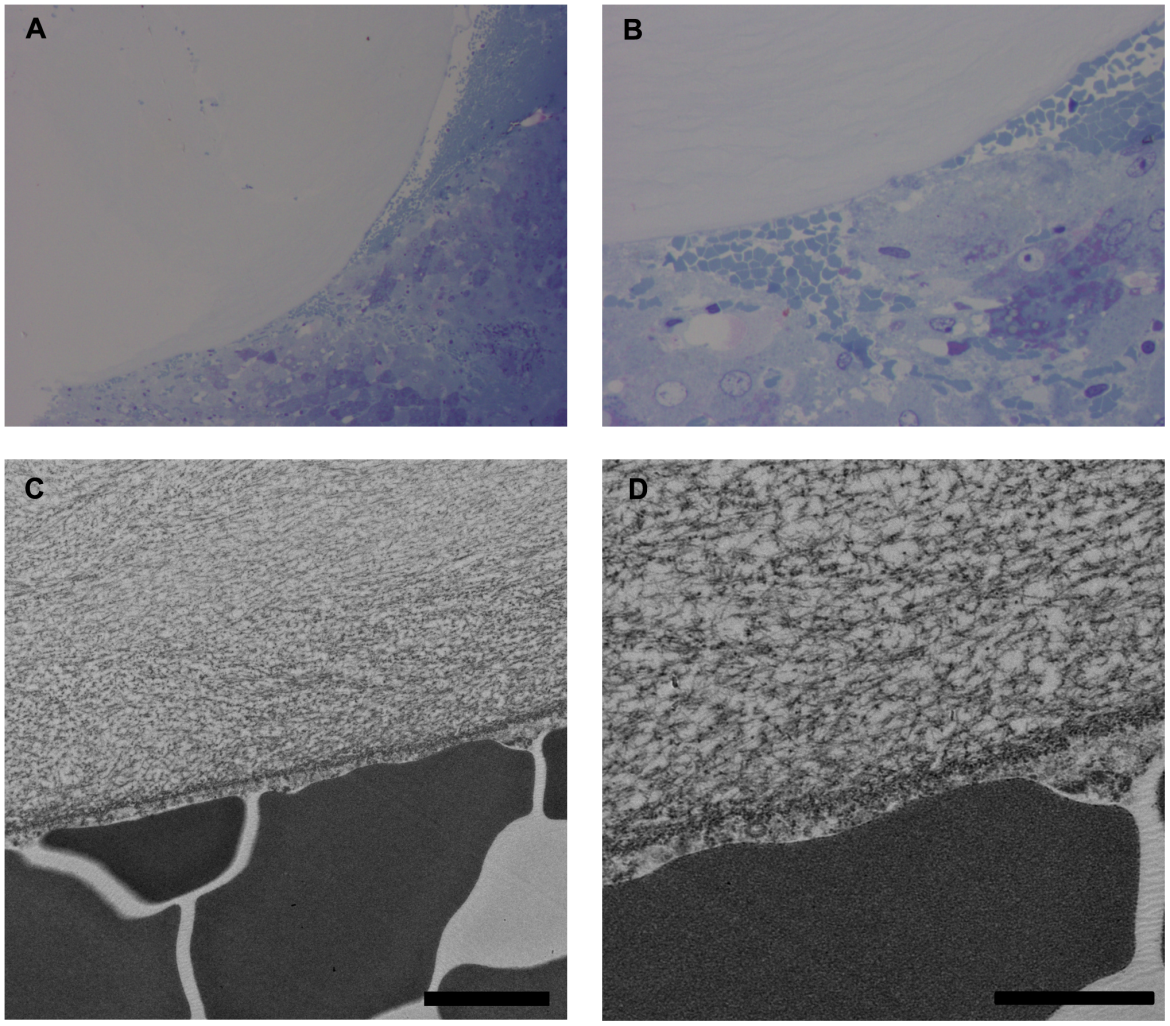
Transmission electron microscopy of the bleeding liver surface. (A, B) Toluidine blue staining was performed to determine the area of interest. SPG-178 was not always in direct contact with the liver. Blood was observed between SPG-178 and the hepatic tissue in most images. Magnifications, 100× (A) and 400× (B). (C, D) Assembled nanofibers of SPG-178 forming a network structure. The density of the nanofibers is extremely densely near the boundary. Scale bars, 2 µm (C) and 1 µm (D).

The boundary between the bleeding surface and the SPG-178 hydrogels was clearly observable by TEM; however, the hydrogels were not always in direct contact with the liver surface. There was evidence of trapped blood between SPG-178 and the hepatic tissue (Fig. 4). Nanofibers of the SPG-178 hydrogels assembled network structures, as expected, and the nanofiber assembly was extremely dense near the interface with the liver surface, although platelet aggregation was not observed. In the RADA16 group, the TEM images were similar to those in a previous study [7]. No differences were observed between the SPG-178 and RADA16 groups.

### Rheology Measurement of Hydrogels

**Figure 5:**
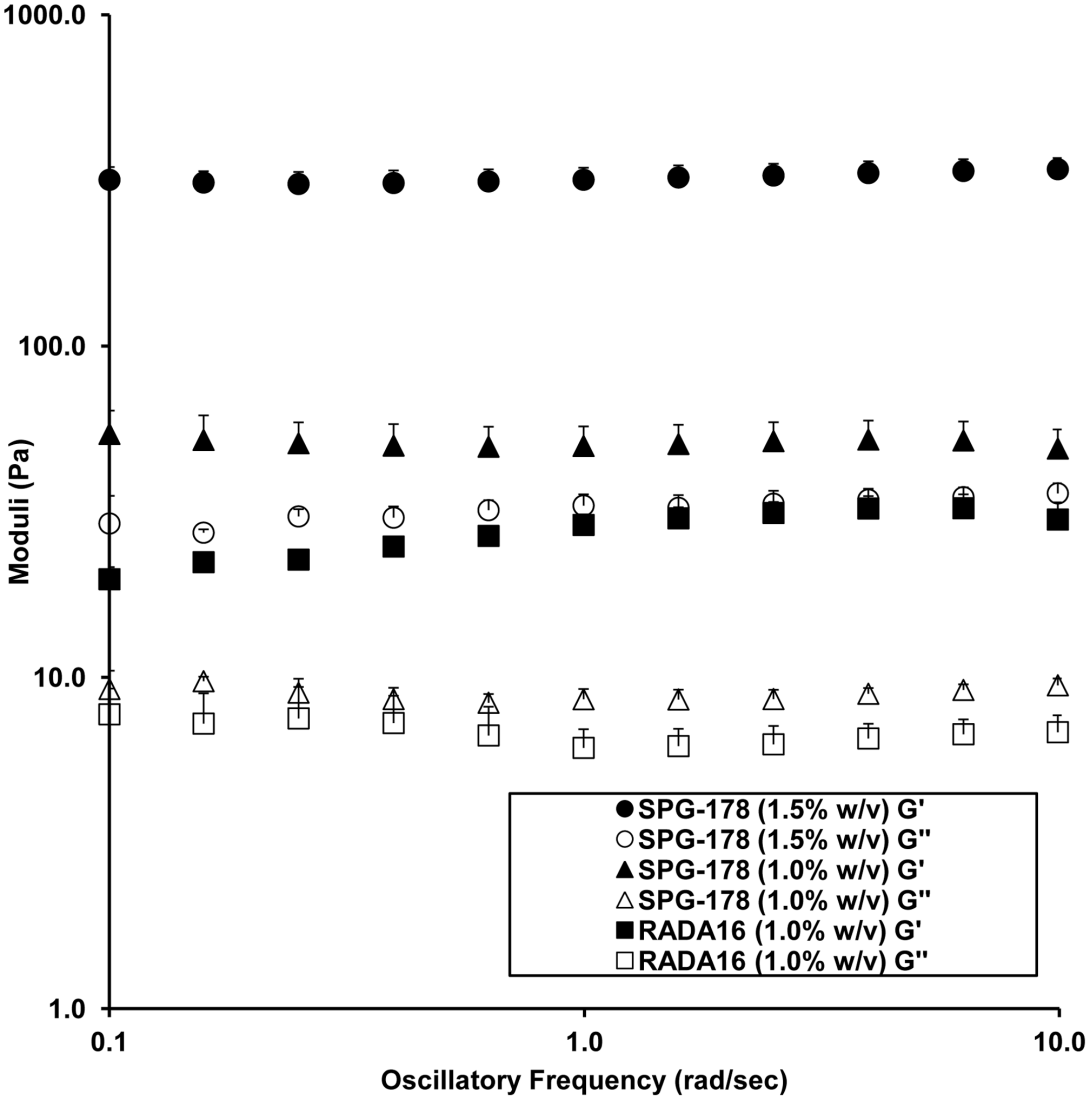
Rheology measurement of the hydrogels. The storage modulus (G′, the elastic response) and the loss modulus (G″, the viscous response) values were relatively constant, and the G′ values were much greater than zero. In addition, the G′ values over the entire frequency range exceeded those of G″. These results reflect the gel-like property of the SPG-178 and RADA16 hydrogels.

Frequency sweep measurements of the SPG-178 and RADA16 hydrogels showed that the G′ and G″ values were relatively constant and that the G′ values were much greater than zero. The G′ exceeded the G″ values over the entire frequency range (Fig. 5). These results reflected the gel-like properties of the SPG-178 and RADA16 hydrogels, with the bleeding duration decreasing as the G′ value increased.

## Discussion

To the best of our knowledge, the present study is the first to report the hemostatic effects of SPG-178 as a highly effective topical hemostatic agent (Fig. 2). SPG-178 has three advantages compared with the conventional topical hemostatic agents: first, it is transparent, thereby allowing the surgeon to visualize the bleeding surface during the hemostasis procedure; second, it is a synthetic material capable of minimizing the risk of unknown infectious diseases associated with biological agents, that may become more important in the future [5], [6], [13]; third, it does not adhere stubbornly to bleeding surface. It is easy to remove if required.

Compared with RADA16, SPG-178 hydrogels showed the shorter bleeding durations. Because of its neutral pH, SPG-178 can be sterilized by autoclaving, which makes the sterilization process simple. The RADA16 hydrogel, in contrast, decomposes with autoclaving [32]. We confirmed that when SPG-178 was prepared under acidic conditions, it also decomposed upon autoclaving. In general, protein bioactivity is often lost with disruption of the tertiary structure. However, a short peptide, such as SPG-178, has no such tertiary structure. For this reason, as long as the covalent bonds of SPG-178, which can be broken by autoclaving in acidic condition, are maintained, it can self-assemble to form a hydrogel. Because there are many cases and conditions where the topical hemostatic agents are required, it is important that the hemostatic procedure is as simple as possible. SPG-178 can be directly applied to the bleeding surface without considering the influence of acidic pH. Thus, SPG-178 hydrogel has a potential to be a ready-to-use topical hemostatic agent when it filled in a proper container. Importantly, in this study, there was no difference in cytotoxicity because of the difference in pH between the SPG-178 and RADA16 hydrogels (Fig. 3). Macrophage infiltration and some foreign body giant cells surrounding both SPG-178 and RADA16 remnants were observed, and these images were similar to those in a previous study [11]. A possible cause for this observation is that the contact time between the liver and hydrogel was short and the remnants were very few. We speculate that differences would have become apparent if the hydrogel had not been quickly removed. Accordingly, we are currently investigating the long-term pharmacokinetics of the SPG-178 hydrogel.

The hemostatic mechanism of these hydrogels has already been reported [7], [11], [12], [13]. In brief, the peptide first comes in contact with electrolytes in body fluids that promote nanofiber assembly and network structure formation, described previously as “multiple-layered fishing nets.” The nets then cover the bleeding surface and prevent the escape of cells and fluid. In this study, application of SPG-178 resulted in an extremely dense assembly of nanofibers on the bleeding surface (Fig. 4). It is already known that the hemostasis is not explainable by clotting or platelet aggregation. Blood clots are produced after injury, but do not begin to form until after 1–2 min [7]. Besides, transmission electron microscopy images showed no evidence of platelet aggregation in this study or in previous reports [7], [11]. Another study reported the antithrombotic effect of L-arginine, that may explain a role of L-arginine residues in SPG-178 and other self-assembling peptide in this effect [33]. We accordingly propose that the hemostatic mechanism of SPG-178 is the same as that of other self-assembling peptide hydrogels. In this study, it was also interesting to note that hemostasis proceeded with time, given that there was no instance of rebleeding when the hydrogels were removed 10 min after application but that rebleeding occurred when the hydrogels were removed immediately after application. We accordingly propose that hemostasis occurred naturally by physiological mechanisms (vessel constriction, platelet formation, and coagulation) during the 10-min interval.

It is important to note the physiological parameters influencing the postoperative hemostatic effect of hydrogels. First, the hydrogel concentration will influence the hemostatic effect, with increased effectiveness as a topical hemostatic agent as the concentration increases [11], [12]. Higher concentrations of the hydrogel allow more rapid formation of a sufficient “fishing net” at bleeding sites. In our study, the mean bleeding duration was shorter in the SPG-178 (1.5% w/v) group than in the SPG-178 (1.0% w/v) group, but the difference was not statistically significant (Fig. 2). However, excessively high concentrations of these hydrogels are not desirable because they are too firm and difficult to handle for such applications. Besides, the product is expensive and technically difficult to synthesize at high concentrations. We accordingly propose that an optimal SPG-178 concentration for use as a topical hemostatic agent is 1.5% w/v. Second, the hydrogel viscoelasticity will also influence the hemostatic effect, with increased effectiveness as a topical hemostatic agent as the hydrogel G′ value increases [12]. The results of our study demonstrated this principle (Fig. 5). The mean G′ value was higher for SPG-178 than for RADA16 at the same concentrations. SPG-178 was much firmer than RADA16. We speculate that a higher viscoelasticity of these hydrogels can stop bleeding more efficiently because they can remain in closer proximity to the bleeding surface. The results thus suggest that the concentration and viscoelasticity of hydrogels are important parameters influencing their hemostatic effects. However, other parameters, including reaction velocity between self-assembling peptides and salt concentrations that may be present in body fluids, may play important roles in these processes.

Our study showed that SPG-178 is a promising topical hemostatic agent. We used a liver laceration model in this study because it is easily reproducible. In previous studies, conventional self-assembling peptide hydrogels showed hemostatic effects in the superior sagittal sinus, spinal cord, artery, and skin [7], [13]. Thus, SPG-178 hydrogel can also be applicable in many surgical procedures.

## Conclusions

We have demonstrated the efficacy of SPG-178, a neutral self-assembling peptide hydrogel as a topical hemostatic agent. The bleeding duration was significantly shorter in the SPG-178 groups than in the RADA16 group. SPG-178 was more effective than RADA16, perhaps owing to the difference in viscoelasticity between the SPG-178 and RADA16 hydrogels. SPG-178 is highly effective and has many advantages: it is non-biological, transparent, nonadherent, neutral, and can be sterilized by autoclaving.

## Supporting Information

Video S1 **Hemostasis in a rat liver laceration model.**
(WMV)
